# Qualitative exploration of the lived experience of adults diagnosed with primary mitochondrial disease

**DOI:** 10.1002/jmd2.12316

**Published:** 2022-07-20

**Authors:** Kathleen D. Valverde, Elizabeth M. McCormick, Marni J. Falk

**Affiliations:** ^1^ Master of Science in Genetic Counseling Program University of Pennsylvania, Perelman School of Medicine Philadelphia Pennsylvania USA; ^2^ Department of Genetics University of Pennsylvania Perelman School of Medicine Philadelphia Pennsylvania USA; ^3^ Mitochondrial Medicine Frontier Program, Division of Human Genetics, Department of Pediatrics Children's Hospital of Philadelphia Philadelphia Pennsylvania USA; ^4^ Department of Pediatrics University of Pennsylvania Perelman School of Medicine Philadelphia Pennsylvania USA

**Keywords:** benefit finding, chronic illness, lived experience, meaning based coping, resiliency

## Abstract

Primary mitochondrial disease (PMD) encompasses a heterogeneous group of energy deficiency disorders that are typically progressive, with affected individuals experiencing an average of 16 multisystem symptoms. Clinical trials are emerging, but current treatment options remain limited. In PMD, the effect of specific disease factors and their relationship to meaning‐based coping has not been studied. Given the connection between prognostic uncertainty and psychological distress in other patient populations, we explored the lived experience of adults with PMD. Adults with PMD caused by pathogenic variant(s) in nuclear or mitochondrial genes impairing mitochondrial function were interviewed. Interview questions addressed the lived experience with PMD, diagnostic journey, practical learnings at the time of diagnosis, suggestions for supportive information to provide at diagnosis, diagnosis impact on daily living and self‐care, and sources of support and hope. Focus group transcripts were analyzed using thematic analysis. Four themes (diagnostic challenges, adaptations to daily living, social implications, and meaning‐based coping) and several subthemes (the importance of being hopeful and benefit finding) emerged. Most participants reported strong family support (9/14) and identified a benefit (9/14) derived from their PMD diagnosis, while (5/14) did not identify any benefits. Benefit finding, reframing, and maintaining a positive attitude emerged as common coping in adults living with PMD. Understanding how adults with PMD cope is essential to provide anticipatory guidance and ongoing support for those struggling with their disease diagnosis, progression, and broader life impact. Our findings suggest that adult PMD patients prefer healthcare providers to inquire about their emotional well‐being and meaning based coping with PMD.

**SYNOPSIS**

Querying primary mitochondrial disease patients about their emotional health, meaning‐based coping, and daily living adjustments may help establish informational and supportive needs.

## 1 INTRODUCTION

Primary mitochondrial diseases (PMD) are highly variable and complex multisystem conditions with a collective minimum prevalence of 1 in 4300 across all ages.^1^ On average, patients experience 16 major clinical symptoms that can significantly limit their ability to complete daily living tasks, work outside the home, and participate in family and community activities.^2^ Significant delays may occur in confirming the specific PMD disease etiology, with more than half of patients reporting having received one or more nonmitochondrial diagnoses before receiving a definite PMD diagnosis.^3^ The heterogeneous nature of PMD makes disease progression challenging to predict, leading to uncertainty and stress in patients and their families. While clinical trials are emerging, current treatment options are limited.^4^ Understanding the lived experience of adults with PMD is essential to providing personalized care for patients and their families. Loss is a significant theme among adults with PMD, where losses are cumulative; for example, job loss leads to loss of social interactions.^5^ PMD is caused by pathogenic variants in nuclear or mitochondrial genes that may arise de novo in affected individuals or be inherited and may follow any inheritance pattern depending on the precise causal gene and/or variant. For families with inherited PMD, issues arise from witnessing the decline of an affected family member, fear of passing the condition onto a child, and fear of the family's future if the affected individual succumbs to their disease.^6^, ^7^

Considering these factors, patients often find coping with a PMD diagnosis challenging. Meaning‐based coping occurs when individuals identify positive meaning from their diagnosis, which is an intrinsic component of resilience.^8^, ^9^, ^10^ Individuals assess their personal beliefs, cultural influences, and goals to motivate their willingness to sustain coping during challenging times. Those with PMD utilize some of these coping mechanisms, including having a “can‐do attitude,” thinking positively, creating hope, taking care of oneself, and using and obtaining social support; however, more support is needed.^5^ The Mitochondrial Disease Community Registry (MDCR) summary report identified three patient needs: enhancing ways to support patients, improving communication, and creating a more trusting relationship between patients and their healthcare providers.^11^ This report identified that patients want healthcare professionals to attend to their psychosocial needs and individualize their care by exploring support strategies and coping with patients and their families.^5^ In *POLG*‐related disease, patients' quality of life was assessed in individuals over 16 years of age, and a decline in mental health and poor quality of life was reported.^12^ Given the connection between diagnostic uncertainty, fear of disease progression, and psychological distress evidenced in this and other patient populations, understanding the lived experiences of adult patients with PMD is essential to evaluate the psychosocial support needs of this population.^5^, ^6^, ^7^

The Mitochondrial Medicine Frontier Program (MMFP) at the Children's Hospital of Philadelphia (CHOP) provides multidisciplinary care and research to improve the health of patients of all ages living with PMD. As part of the program's mission to improve the quality of life of PMD patients, we conducted exploratory qualitative interviews to understand the needs of adult patients. Through analysis of themes raised in these focus groups, we sought to explore the lived experience of adults with a definitive PMD diagnosis, better understand the broader disease impact on their quality of life and develop enhanced support services for PMD patients and families.

## 2 METHODS

### 2.1 Study design, sampling, recruitment, and participant description

Fifty adults with PMD, genetically confirmed by the presence of a pathogenic variant in either mitochondrial DNA (mtDNA) or nuclear DNA (nDNA) impairing mitochondrial function, were recruited from the Children's Hospital of Philadelphia Mitochondrial Medicine Frontier Program (MMFP) to participate in an IRB reviewed exempt qualitative study (CHOP IRB#19‐015957). By design, focus groups were kept to 2–4 participants to encourage active participation. Interviews were conducted online by video conferencing. Participants had the option of selecting audio‐only or audio and video connections. Verbal consent was obtained at the start of the sessions, and only first names were used to maintain patient confidentiality.

### 2.2 Data collection

Two licensed genetic counselors (KDV and EMM) facilitated the discussions using an investigator‐designed semistructured interview guide composed of five questions and three prompts developed per literature review of the experiences of adults with chronic progressive disease and clinicians who care for adults with PMD (Appendix A). Questions were designed to explore the impact of progressive disease on daily life, information received at the time of diagnosis, self‐care issues, sources of support, and possible benefits derived from the diagnosis, including sources of hope. Video recordings were transcribed verbatim.

### 2.3 Data analysis

**FIGURE 1:**
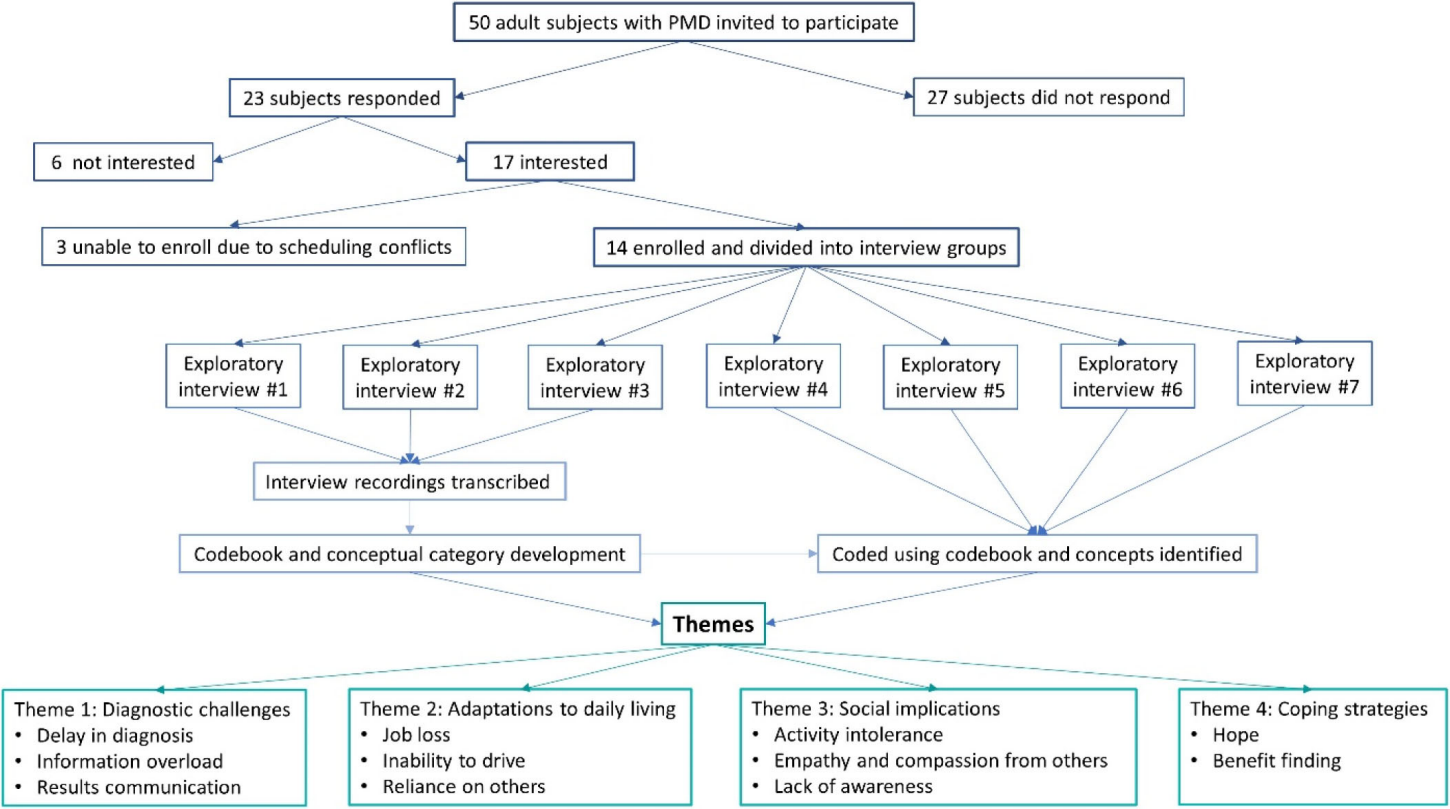
Study design and identification of themes. Fourteen subjects were divided into seven groups to undergo exploratory interviews. Transcripts from these interviews were analyzed and coded and study enrollment stopped when theme saturation was reached.

Exploratory interviews were reviewed using qualitative descriptive and thematic analyses. Using the first three interview transcripts, two researchers (KDV and EMM) used constant comparative analysis for coding and conceptual category development.^13^ Concepts were identified, and codes were assigned, including subthemes by one researcher (KDV) using NVIVO‐12 software. A second coder (EMM) reviewed the data for reliability. Subsequent interviews were coded using the same process. Meetings between researchers (KDV and EMM) were held regularly to determine if new categories were emerging or if saturation had been reached.^14^ After the seventh interview (*n* = 14 study participants), no new themes were emerging, suggesting that saturation was reached, and recruitment was suspended (Figure 1).

## 3 RESULTS

Recruitment emails were sent to 50 potential patients (4 Hispanic, 2 African American, 44 White), and 14 agreed to participate (30%, all White). Seven focus groups were conducted virtually between October 2019 and July 2020, with a total of 14 participants (9 women and 5 men) between the ages of 24 and 69 years. All participants were white, non‐Hispanic, and manifested clinical symptoms of PMD. Six participants were parents, and eight did not have children. Of the eight participants without children, two were women contemplating pregnancy but had concern for their health and the child's risk of inheriting PMD (Table 1). All fathers had healthy children, and all mothers had at least one affected child (all with an mtDNA etiology). Participants answered all questions and prompts. Interviews ranged between 50 and 90 minutes. The PMD genetic diagnoses of the participants are listed in Table 2, including 11 individuals with mtDNA genetic etiologies and three individuals with nDNA genetic etiologies.

**TABLE 1:** Participant demographics

*Gender*		
Male		5
Female		9
*Age (years)*		
20–29		3
30–39		4
40–49		2
50–59		3
60–69		2
*Relationship status*		
Males	Married	3
	Single	2
Females	Married	4
	Committed relationship	2
	Single	3
*Parenthood*		
Males	Fathers	3
	Childless	2
	Considering children	0
Females	Mothers^a^	3
	Childless‐considering pregnancy	2
	Childless	4
*Ethnicity*		
White non‐Hispanic		14

*Note*: Median age was 41 years, and range was 25–69 years.

^a^ None of the mothers were considering pregnancy.

**TABLE 2:** Genetic diagnoses of participants

Genetic etiology	Number of individuals (participant number)
m.3243A>G (*MT‐TL1*)	4 (4, 5, 13, 14)
m.7471dupC (*MT‐TS1*)	1 (11)
m.7497G>A (*MT‐TS1*)	1 (3)
m.8344A>G (*MT‐TK*)	1 (6)
m.9026G>A (*MT‐ATP6*)	1 (1)
Single large‐scale mitochondrial DNA deletion (SLSMD)	3 (7, 10, 12)
*ACAD9*	1 (9)
*DLD*	1 (8)
*POLG*	1 (2)

**FIGURE 2:**
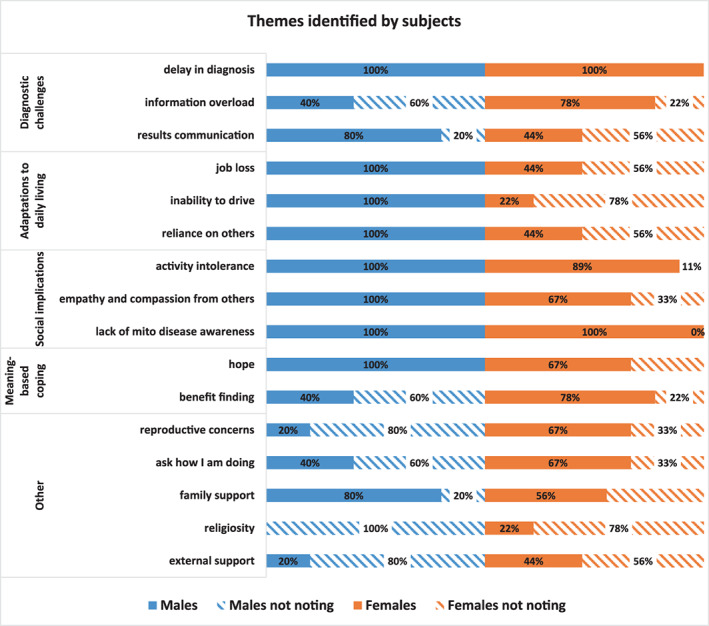
Themes identified by adult PMD study subjects (*N* = 14, 5 male, 9 female). The solid color bar indicates the percent of males (blue) or females (orange) who discussed the theme during the interview. The hashed bar indicates the percentage of males (blue) or females (orange) who did not bring up the theme during the interview.

The participants interviewed varied in their age of diagnosis, time living with PMD, geographical location, and disease progression. Despite the demographic diversity of subjects, four themes emerged in reviewing the interviews, including diagnostic challenges, adaptations to daily life, socialization implications, and meaning based coping. Therefore, the results were organized by these four main themes (Figure 2). Additional themes of reproductive concerns, psychosocial checking‐in, family support, and external support also emerged.

## 4 THEME I: DIAGNOSTIC CHALLENGES

### 4.1 Subtheme I‐1: delay in diagnosis

All participants relayed frustration with the time it took from symptom onset to a clinical definitive PMD diagnosis. They share dissatisfaction with many healthcare providers seen before a definitive diagnosis was confirmed. Many reported that their symptoms were dismissed or not taken seriously along their diagnostic journey (Table 3, subtheme I‐1).

**TABLE 3:** Illustrative quotes from focus discussion groups

Subtheme number	Subtheme	Illustrative quotes	*n*
**What information was most helpful to receive at the time of your diagnosis?**			
**I. Diagnostic challenges**			
1	Delay in diagnosis	*“No one had an explanation. I was going down the wrong path for a long time…It (diagnosis) does not explain all the symptoms but makes a lot more sense than it did early on, so the diagnosis was a long time coming.” (Participant 13)* *“It took a long time to receive the diagnosis. I had been complaining of symptoms in elementary school, a lot of being told that there was nothing wrong with me and that my testing was normal. I was really happy to have a name for it, but I think that my biggest disappointment came when I realized that there is nothing that I can do to fix it.” (Participant 5)*	14
2	Information overload	*“There was a lot of when you grow up; you cannot do this…You're going to have a shorter lifespan and…. a lot of talk about things decades ahead. Much of what was said did not happen. Telling a child that is not ideal and probably causes a lot of trauma and psychological impact.” (Participant 10)* *“Now I am 57 years old. At 15, I was told that this disease has no treatment or cure. I was looking at a life of being debilitating as I aged. It was devastating.” (Participant 8)*	9
3	Results communication	*“A nurse called me on the phone, you have mitochondrial disease. I would have liked to been called probably into the doctor's office or maybe a more formal way of telling me and just giving me more information. It was kind of like almost nonchalant, a phone call. Okay, I've got mitochondrial disease and the follow up with the doctor was a month or two later, so you are Googling and Web MD and all this stuff, trying to figure out what the heck mitochondrial disease is, so I think that was not helpful.” (Participant 2)* *“I feel sorry for the neurologists, to handout this diagnosis… sorry, buddy, there is not much we can do for you. So I have to cheer myself up by accepting where I am and trying not to regret what I do not have.” (Participant 3)*	13
**Since your diagnosis how has your life changed?**			
**II. Adaptations to daily living**			
1	Job loss	*“I was a staff social worker. It was a lot; I wound up getting pneumonia twice. It (leaving my position) was a difficult decision, mostly because my symptoms were coming on, the muscle weakness.” (Participant 6)* *“I work 3 days a week now. I'm talking and then I take a break just to conserve my energy. By the end of my three consecutive days. I am exhausted. I am homebound, I have no energy to do other things. So I found, especially over the last couple years. I'm spent.” (Participant 10)*	9
2	Inability to drive	*“Probably the biggest thing is not being able to drive any longer. Well, my car is paid off and then all of a sudden it was like, you cannot drive. It was difficult to be 33 years old and not able to be independent and to be dependent on someone else to get around.” (Participant 12)* *“I cannot drive anymore. I mean, I can, but I probably would hurt somebody. I have a history… of not paying attention while I'm driving. I managed to total about eight cars, nobody really got hurt too bad. A couple of broken bones here and there, but it's not a good idea when I have that much trouble driving when I had all my capacity about me, now I have very little of it. I pretty much surrendered the keys.” (Participant 3)*	7
3	Reliance on others for care	*“Even though my parents, without question, welcomed me back into their home. I'm sure they never envisioned at the age 70 they would have their adult son living back in their house. Basically, I'm totally relying on them for transportation and getting me groceries and, you know, things like that.” (Participant 2)* *“(I have help) …from cooking and cleaning to everything else. It's good and bad. Sometimes you like being alone, and other times you are glad to be in the same space.” (Participant 6)*	9
**Tell us how you care for yourself and get support.**			
**III. Social implications**			
1	Activity intolerance	*“Hanging out with a group of people, I get fatigued, not just mentally but like I mean physical fatigue. You know having fun is just kind of mentally exhausting keeping up with a conversation and things like that. It's just draining to me where you think it would be fun, and it is, but at the same time, it might take me, a couple of hours after that to recover or the next day. I'm just feeling weak with some enjoyable things.” (Participant 2)* *“I guess, what probably bothers me the most is just a general achy malaise feeling, and it comes and goes. Morning is my best time ‐ my battery recharges overnight ‐ mornings I do well. I do not know when or if I will start feeling bad. It could be at noon; it could be in the evening. The unpredictability is something that really bothers me.” (Participant 11)* *“Yesterday my wife and I went out… It's so weird. I do not feel part of it anymore. You know, the whole world but it's outside and cool, and it smells nice… Cars and trucks are driving all‐around, living their lives. I'm just like sitting in the passenger seat. It's like watching TV. So it's affected me that way negatively, you know. I have to make an effort to get up some days.” (Participant 3)*	13
2	Empathy and compassion from others	*“I think an interesting experiment would be to have mito patients say that they have mitochondrial cancer; what would the reaction be? What would that mean for people, and… what you are going through.” (Participant 5)* *“For the most part people are pretty understanding now that I walk with a cane, it's a visual cue to people. It makes it obvious that I've got difficulties. Early on people cannot look at you and know that you have trouble with your vision or whatever. They were not quite as sympathetic. So as things progressed… as things got worse, people were more understanding.” (Participant 11)*	11
3	Lack of general knowledge and understanding of PMD	*“I go to the dentist and they are intrigued by my mitochondrial disease, everyone is. I mean, it's 2020, and I was diagnosed in the 1990s and it still has not changed in terms of knowledge.” (Participant 10)* *“Yes, the literature is growing, and there's a lot more (information) than there was in the 80s. It's (PMD) still rare compared to say diabetes. We're all still trying to understand.” (Participant 9)*	14
**What gives you hope? Can you identify any positive changes/ benefits since your diagnosis?**			
**IV. Meaning based coping**			
1	Hope	*“HOPE stands for (H) holding on to faith in religion, yourself and other people…(O) opportunities… maybe my phone will ring today and bring an opportunity, (P) …process what you have learned today. Today, I'm reflecting on her story and what she has for her self‐care and what I've taken from that. That's really cool to me, and perhaps she can learn from mine (story). (E) encourage the soul.” (Participant 9)* “*If I take it one day at a time it is okay. I am hopeful that I am on this mito cocktail. I'm hoping that the arginine will help with my migraine. I mean, those are like the little things. My silver linings.” (Participant 13)*	11
2	Benefit finding	*“As a male, I cannot give it to my children. Knowing that is probably the only beneficial thing that I know about the diagnosis. That helps me a bit.” (Participant 4)* *“I like to fix things and it's moved from fixing cars and the house to fixing scooters ‐ something more manageable. I still have a lot of the same interests, but I just realized that I cannot do things and try to accommodate the new.” (Participant 11)* *“The severity of mine is stable. I think I'm very blessed. I'm still able to do pretty well with self‐ care and my needs. It just that my arms get tired when I'm washing my hair. But to me, that's my normal.” (Participant 8)*	9

### 4.2 Subtheme I‐2: information overload

As participants recalled the disclosure of their diagnosis, information overload was a common theme, “*I mean it's kind of a fire hose of information early on*” (Participant 11). They recalled feeling overwhelmed with the extent of information shared. The medical terminology was confusing and unfamiliar to many (Table 3, subtheme I‐2). Participants expressed being emotionally distraught and recalled that it was difficult to comprehend all the information conveyed. One woman who was diagnosed along with her son shared,

*“It's still a hard process, and I can't tell you the names of my mutations. I can't tell you all that stuff… I kind of block out the medical end of it. You know, unless you can dumb it down, great, really, for me, it was a lot to absorb, and still is” (Participant 1)*.

### 4.3 Subtheme I‐3: results communication

Disappointment with healthcare providers' sensitivity when disclosing a diagnosis emerged. One participant described the nurse who shared his serious medical condition as ‘nonchalant.’ Adults diagnosed as children or teenagers expressed how difficult it was to learn that they had a progressive, debilitating disease (Table 3, subtheme I‐3). They shared how challenging it was to process this information and its significant toll on their emotional well‐being. The negative information shared was devastating to hear during their formative years. Some patients experienced slower disease progression than was predicted at their diagnosis, leading to the reclassification of their PMD at a later age.

*“Doctors told me that I would die in my teens and thinking back a long time ago… that was traumatic for me. I am still here, and who knows how long it is going to be, but I think that whether it's an age or a symptom, that we need not put limitations, or symptoms or expectations on children or people” (Participant 9)*.

## 5 THEME II: ADAPTIONS TO DAILY LIVING

Many participants shared that their most significant daily living adjustments were their inability to work outside of the home (9/14) and losing their ability to drive (7/14). More than half of the interviewees (9/14) discussed their reliance on others for self‐care, financial assistance, and emotional support.

### 5.1 Subtheme II‐1 job loss

*“I stopped working. I also had to give up driving. And so my world kind of shrunk down to a ride on my scooter. I am kind of housebound, I don't leave a lot” (Participant 2)*.

All men interviewed (5/5) had advanced disease and were unable to work due to fatigue and inability to maintain job responsibilities. The women interviewed were more variable in age and job status. The younger women (ages 20–29) worked outside the home or cared for their families (5/9). To continue working, one woman shifted to part‐time hours and moved to reduce commuting time (Table 3, subtheme II‐1). The other women (4/9) could no longer work due to their health concerns. Participants reported that job loss led to feelings of social isolation.

### 5.2 Subtheme II‐2: inability to drive

Dependency on others for transportation forced a lifestyle that the participants did not have the opportunity to choose. Participants spoke about losing spontaneity and the need to schedule travel in advance. Many participants noted safety as a concern when they decided to discontinue driving. Two participants stopped driving after being involved in car accidents (Table 3, subtheme II‐2).

### 5.3 Subtheme II‐3: reliance on others

More than half of the interviewees (9/14) discussed reliance on others for self‐care, financial assistance, and emotional support. Most received care from family members, but one participant needed in‐home health care. Two adults decided to move back in with their parents and noted that the loss of independence was a difficult adjustment. One woman shared losing her boyfriend and dog when moving back with her parents (Table 3, II‐3 subtheme).

## 6 THEME III: SOCIAL IMPLICATIONS

### 6.1 Subtheme III‐1: activity intolerance

Almost everyone (13/14) discussed how fatigue negatively impacted their daily life and led to socialization challenges by limiting their ability to participate in social activities. Participants described having to choose what activities to engage in based on how they feel each day, leading to last‐minute cancelations. This was acknowledged as causing disappointment for the individual and friends and relatives who expected them to participate in a social event. This uncertainty was described as frustrating and made planning difficult. Participants discussed the need to recover after participating in activities, making it difficult to engage in enjoyable activities such as keeping up with conversations (Table 3, subtheme III‐1).

Participants shared that friends and relatives did not understand their need to rest after a busy day. One shared her experience of being teased,

*“I just have to lay in bed for a full day, and then that ruins plans with the family. I'm at a campsite in bed. People make fun of me and say, oh, you slept in today. I blame it on migraines, but it's just my energy. I don't want to get out of bed because I did too much the day before (Participant 1)*.

Even within families with multiple affected individuals, there can be a lack of understanding, illustrating how normative expectations around energy and productivity get internalized. This mother reflected upon her reaction toward her son, regretting her judgment of his limited energy,

*“Oh, you had a spurt of energy to go on X‐box. Why didn't you have that energy to get to school… I catch myself doing it with him, and it doesn't make any sense” (Participant 1)*.

Participants reported that social isolation or feeling dissociated from the world could lead to disengagement, causing psychological distress (Table 3, subtheme III‐1).

### 6.2 Subtheme III‐2: empathy and compassion from others

Most participants (11/14) described their mitochondrial disease symptoms of illness as “invisible” since there are often no outwardly visible signs. It was not until they used “helpers,” assistive devices such as walkers or canes, that they received immediate empathy and concern. One patient commented that there is no instant recognition when explaining that she has mitochondrial disease and wants to call it “mitochondrial cancer” to elicit more empathy and support (Table 3, subtheme III‐2).

### 6.3 Subtheme III‐3: lack of general awareness of primary mitochondrial disease

All participants (14/14) shared their frustrations with the general population's lack of understanding of PMD. Older patients expressed frustration that there is still a limited understanding of PMD in the medical community decades after their diagnosis (Table 3, subtheme III‐3).

## 7 THEME IV: MEANING BASED COPING

### 7.1 Subtheme IV‐1: hope

All participants answered the question, “What gives you hope?”. Their responses included knowing that their disease is stable, anticipation of new treatments and cures, and the potential every day brings. The overarching theme among the participants was to stay positive and be hopeful. One participant used HOPE as an informative acronym to cultivate the emotion (Table 3, subtheme IV‐1).

### 7.2 Subtheme IV‐2: benefit finding

In parallel to hope, nine participants articulated benefits realized from their diagnosis (Table 3, subtheme IV‐2). Of those who did not identify a benefit, two were newly diagnosed, two had advanced disease, and one struggled with her reproductive options. All three fathers said that not passing PMD on to their children (as they had mtDNA genetic etiologies of their disease) was a benefit. Other benefits included developing greater empathy for themselves and their children, sharing family time, and accepting assistive devices to increase mobility. Half of the participants (7/14) explained that embracing “helpers” was a process they now view as beneficial. Subjects reported that ‘helpers’ enabled them to conserve energy to participate in other, more enjoyable activities. Participants spoke about learning to reimagine their daily activities to save their energy while still enjoying hobbies and interests. One man shared this benefit,

*“My parents welcomed me back into their home, they are in their 70s and probably never felt that they would have their adult son back with them, but we're sort of blessed, back together. I can enjoy being with them in their retirement” (Participant 2)*.

## 8 ADDITIONAL FINDINGS

### 8.1 Reproductive concerns among women with PMD

While not prompted, two‐thirds (6/9) of the women interviewed discussed the impact of PMD on their reproductive choices. Four themes were identified, including (1) foregoing parenthood due to their diagnosis, (2) guilt from passing the condition to their child, (3) availability of assistive reproductive technologies, and (4) an overall sense of loss. One mother spoke about her guilt,

*“I just began seeing a therapist recently because I feel a lot of guilt. That I passed this on to my son, now that I'm talking to somebody, I hadn't realized how bad I felt, guilty” (Participant 1)*.

Two younger women (ages 20–29) were concerned about the limited reproductive options available. Three women shared that they were discouraged from having children.

*“My diagnosis is a large part of why I don't have children. …My doctor was worried about the stress on my body. He flat out said, “Don't have children." For me, I didn't want to do that to my child" (Participant 7)*.

### 8.2 “Please ask me how I am doing”

The majority of the adult PMD study participants were appreciative to be asked about their emotional well‐being. One stated the importance of hearing the patient's perspective: “*I think it's great to learn from the patient, you know, the patient's voice and all this. I think it's a big thing for doctors and clinicians to understand*” (Participant 2). Participants expressed that it would be helpful to be asked about their emotional well‐being during routine appointments. At the end of an interview, one participant explained

*“This conversation is a conversation that I never had since my diagnosis. We talk a lot about physical symptoms… at appointments. It's always muscle function and energy, and this diagnosis impacts people for the rest of their life, and it's not something ever discussed, and that should have been a part of my treatment as well. Those conversations are super emotional. I have had this for two decades now, and it's still something I've clearly not processed, and that should have been a part of my treatment as well. That can make things a little more positive for people diagnosed and…by asking patients about their mental states and how it affects their lifestyle, psyche, and mood, and then addressing it. I think that is important.” (Participant 8)*.

### 8.3 Family support

Most participants reported benefitting from strong family support (9/14). One shared, *“Family is really important because other people can't really provide you with the help you need” (Participant 3)*.

### 8.4 External support

Only two participants reported being religious. Engaging in online support varied from active to occasional to no use. One participant explained her feeling regarding obtaining support services,

*“(Getting support)… it's something that I have not done, and I don't know why I haven't connected with others, it's always been in the back of my mind. I'm going to get more involved. You're probably the first person I've ever seen with mito disease except potentially in a waiting room. It's (this conversation) giving me the motivation to start finding people like myself, that sounds so good. Not currently involved, but definitely, something I will start to do” (Participant 10)*.

The overall sentiment among the participants was that listening to others' perspectives was a valuable experience.

## 9 DISCUSSION

Focused exploratory interviews conducted with 14 adults affected with PMD revealed that benefit finding, reframing, and maintaining a positive attitude were commonly utilized meaning based coping skills. Notably, adult PMD patients preferred healthcare providers to inquire about their emotional well‐being and coping with PMD specifically. The Mitochondrial Medicine Society acknowledges the higher prevalence of psychiatric disorders in individuals with PMD and recommends utilizing a standardized tool for depression and anxiety to be administered regularly, although a specific tool has not been recommended.^15^ These concerns could be assessed by a structured interview guide or validated surveys such as those assessing quality of life, coping, or resiliency. These could be administered  during a patient visit and the practitioners could review the responses allowing them to offer targeted referrals and anticipatory guidance that align with the patients' needs. Other disease organizations such as the National Multiple Sclerosis Society (NMSS) have guidelines suggesting yearly emotional well‐being assessments. The NMSS website homepage has a resilience portal with several patient resources, including a video, “Resilience: Addressing the Challenges of MS,” and a discussion guide that provides example topics to discuss with healthcare providers.^16^ Mitochondrial Disease supporting organizations could create similar patient resources.

The findings in this study are similar to what has been reported in the few published studies focused on the long diagnostic odyssey often experienced before receiving a definitive confirmed diagnosis of PMD.^5^, ^6^, ^7^, ^17^ Our participants report being overwhelmed at the time of diagnosis and learning about long‐term complications that may be years away. These participants expressed their preference to live in the present and learn about complications as they arise and their disease progresses. Furthermore, they were looking for guidance on sharing their diagnosis with others. Healthcare providers could provide patients with specific language explaining their symptoms' unpredictable nature and limited energy.

Finding novel ways to help adults with PMD adjust to their diagnosis and disease progression can provide more hope for patients living with progressive disease. The MDCR summary report showed patients are hopeful about their future despite many challenges.^11^ Hope has been studied in patients with multiple sclerosis and it was found that hope does not necessarily mean hope for an immediate cure but rather knowing that research continues. Patients with MS expect their neurologists to update them on new research developments.^18^ Healthcare providers should be encouraged to share the same updates with PMD patients.

The lack of awareness of PMD among healthcare providers and the general population remains an ongoing challenge experienced by adult PMD patients, demonstrating the need for continued disease‐specific organizations' efforts to increase awareness and understanding of PMD.^19^ Given the discrepancy between what providers detail at the time of diagnosis and the struggles that subjects with PMD voiced, providers should consider calibrating conversations around diagnosis and management to the patients' age and stage of the disease.

Our findings suggest that the lack of understanding about PMD leads to patient frustration and confusion among friends and family, who often do not understand their fatigue and inability to plan and participate in social activities. Healthcare providers could better guide and prepare newly diagnosed patients to communicate their new diagnoses to others by providing a new patient guide with specific language and strategies around disclosing a diagnosis, including how to explain the unpredictable nature of their symptoms that may be progressive over time as well as their limited levels of energy. A new patient guide for MS patients is available on the NMSS website.^16^ Mitochondrial Disease support organizations could develop similar materials.

Adjustment to a PMD diagnosis is complex, and adult PMD patients experience diverse losses leading to socialization challenges.^5^ Developing strategies for coping with these losses is critical as coping is necessary for resilience, the positive response to a stressor.^20^ Coping differed among participants, with some individuals having innate coping skills. However, it is important to recognize that meaning based coping can be fostered and learned.

We specifically asked what meaning patients have derived from their diagnosis because meaning‐focused coping is necessary to develop resilience. An essential aspect of benefit finding is reordering priorities by prioritizing achievable tasks.^21^ When adults with PMD assess their daily activities in light of their disease progression, this allows them to conserve energy for more quality family time, a benefit described by several individuals. Accepting one's limitations and the need to modify one's daily life takes time and significant adjustment. Benefit finding develops over time and can be a way to reframe living with a progressive condition. Studies exploring benefit finding revealed that its positive effects take time to be realized, where a minimum of 2 years from the initial stressor is necessary.^22^ Genetic counselors are skilled at discussing transitional life events, prognosis, reproductive concerns, and familial implications of genetic disease with patients. This comprehensive genetic counseling model has been reported in the Friedreich ataxia patient population.^23^ Genetic counselors may help adults with PMD who feel isolated and unprepared to adapt to their diagnoses' wide‐reaching effects on their lives.

Social isolation among adults with PMD is concerning. Healthcare providers often recommend that patients consider online support. However, few in our cohort were consistently using online support services. Future investigations would help understand the potential influence of PMD symptoms such as impaired hearing, vision, energy, or motor function on their uptake of online resources. Previous research has identified a lack of supportive services for adult patients with PMD.^19^ During our interviews, it became apparent that participants enjoyed hearing each other's stories. One remarked that a buddy system might offer comparable benefits to the interview on a longer‐term basis and encourage patients to participate in online virtual support or in‐person support groups to share their experiences and meaning‐based coping.

Reproductive options for women with PMD due to mtDNA pathogenic variants remain limited, and participants in this study endorsed frustration over the limited options available to them and the significant effect their disease has on their family planning.^24^, ^25^ It is essential that healthcare professionals query patients about reproductive concerns and ensure genetic counseling is recommended, so patients have the most updated information to guide family planning decision‐making.^24^

### 9.1 Strengths and limitations

We aimed to explore adult patients' lived experiences with genetically confirmed PMD to identify common themes using an exploratory qualitative design. However, this study's limitations are small sample size and a homogenous group recruited from one healthcare institution. The contribution of mood disorders to a patient's experience of PMD was not evaluated. Indeed, while depression, anxiety, and other mood disorders are common in patients with PMD, study participants' mental health history was only identified in four patient medical records and not explicitly documented in the other patient records. Lastly, results from sufficiently well‐connected individuals to reach out and engage over teleconference for the interview may not be generalizable to nonresponders, given this study's 30% response rate.

## 10 CONCLUSIONS

Expanded genome‐wide testing for both mtDNA and nDNA genetic causes of PMD continues to increase the number of patients diagnosed, including those with atypical and milder presentations. Healthcare providers need to be prepared to address patients' long‐term needs, including their emotional well‐being. We recommend that healthcare providers share information about patient advocacy and support groups and consider referring PMD patients who need additional support for mental health services. Continued research on coping mechanisms such as benefit finding is necessary to validate and further expand upon these findings. Future research includes validating these findings in a larger sample of PMD patients, potentially through a survey study to identify validated coping and resilience assessment tools that would be relevant to the PMD community. In addition, finding ways to better understand and support reproductive decision‐making in adult women with PMD is needed.

## AUTHOR CONTRIBUTIONS

Marni J. Falk provided research program oversight and regulatory approval (CHOP IRB #08‐6177 and #19‐015957). Kathleen D. Valverde and Elizabeth M. McCormick created the exploratory interview guide, facilitated enrollment, and conducted the exploratory interviews. Kathleen D. Valverde performed thematic analysis with Elizabeth M. McCormick as second coder. Kathleen D. Valverde, Elizabeth M. McCormick, and Marni J. Falk wrote the manuscript and approved the final version.

## FUNDING INFORMATION

This work was funded in part by the Children's Hospital of Philadelphia Mitochondrial Medicine Frontier Program.

## CONFLICT OF INTEREST

Kathleen D. Valverde and Elizabeth M. McCormick has no conflicts of interest to report. Marni J. Falk is engaged with several companies involved in mitochondrial disease therapeutic preclinical and/or clinical stage development not directly related to the work, including as an advisory board member with an equity interest in RiboNova, Inc., scientific board member as a paid consultant with Khondrion and Larimar Therapeutics, as a paid consultant (Astelllas Pharma Inc., Casma Therapeutics, Cyclerion Therapeutics, Epirium Bio, HealthCap VII Advisor AB, Imel Therapeutics, Minovia Therapeutics, NeuroVive Pharmaceutical AB, Reneo Therapeutics, Stealth BioTherapeutics, Zogenix Inc.) and/or as a sponsored research collaborator (AADI Bioscience, Astellas Pharma Inc., Cyclerion Therapeutics, Epirium Bio (formerly Cardero Therapeutics), Imel Therapeutics, Khondrion, Minovia Therapeutics Inc., Mission Therapeutics, NeuroVive Pharmaceutical AB, PTC Therapeutics, Raptor Therapeutics, REATA Inc., Reneo Therapeutics, RiboNova Inc., Standigm Inc., and Stealth BioTherapeutics). MJF also receives royalties from Elsevier, speaker fees from Agios Pharmaceuticals, and an educational honorarium from PlatformQ.

## ETHICS STATEMENT

All procedures followed were in accordance with the ethical standards of the responsible committee on human experimentation (institutional and national) and with the Helsinki Declaration of 1975, as revised in 2000.

## INFORMED CONSENT

Informed consent was obtained from all patients included in this study.

## ANIMAL RIGHTS

This article does not contain any studies with animal subjects performed by any authors.

## Data Availability

The data supporting this study's findings are available on request from the corresponding author, KDV. The data are not publicly available due to their containing information that could compromise the privacy of research participants.

## EXPLORATORY INTERVIEW GUIDE QUESTIONS

1. Please tell us about how receiving your diagnosis may have impacted your daily life.
  - Probe‐ your family?
2. What information was most helpful for you to receive at the time of your diagnosis?
3. Since your diagnosis how has life changed?
  - Probe‐ can you identify any positive changes/ benefits?
4. Tell us how you care for yourself and get support.
  - Probe‐ what gives you hope?
5. Knowing what you do now, what advice would you give to someone newly diagnosed with PMD and their family?
